# Motor and Physical Function Impairments in Middle-Aged and Older Adults in the Baltimore Longitudinal Study of Aging

**DOI:** 10.1093/geroni/igaa057.748

**Published:** 2020-12-16

**Authors:** Yurun Cai, Qu Tian, Yuri Agrawal, Eleanor Simonsick, Jennifer Schrack

**Affiliations:** 1 Johns Hopkins Bloomberg School of Public Health, Baltimore, Maryland, United States; 2 National Institute on Aging, Bethesda, Maryland, United States; 3 Johns Hopkins University School of Medicine, Baltimore, Maryland, United States

## Abstract

Older adults experience motor function decline early in the disablement process, impacting daily activities and contributing to adverse health outcomes. Few studies have comprehensively examined the interrelationships among motor and functional impairments and investigated whether their contributions to mobility difficulty vary in well-functioning older adults. We examined direct and indirect associations of motor and physical function impairments with slow gait speed (<1.0m/s) and mobility difficulty using structural equation modeling (SEM) among 858 participants aged ≥50 years in the BLSA (mean age=74.1±10.6, 55% women). Motor and physical function tests included grip strength, knee extension strength, proprioception, finger tapping, standing balance (semi-, full-tandem, single-leg), repeated chair stands, and usual gait speed. Mobility difficulty was defined as self-reported difficulty in walking ¼ mile or climbing stairs. Motor and physical function impairments increased linearly with age, with 27.6% of participants having slow gait speed and 10.4% having mobility difficulty. Age-adjusted SEMs identified chair stands pace as the strongest predictor of slow gait speed, followed by latent factors of upper and lower extremity muscle strength and standing balance. Chair stands pace was the strongest predictor of mobility difficulty, followed by gait speed. Latent factors of muscle strength, proprioception, finger tapping, and standing balance were indirectly associated with mobility difficulty via gait speed. All models showed good model fit (RMSEA<0.05, CFI>0.95). These findings suggest components of strength and balance are among the most important contributors to poorer functional performance in mid-to-late life. Future longitudinal studies gauging the effect of change in these factors are warranted.

